# Guided Trocar Insertion in Highly Myopic Eyes

**DOI:** 10.1097/IAE.0000000000003997

**Published:** 2024-04-18

**Authors:** Alfonso Savastano, Patrizio Bernardinelli, Umberto De Vico, Giulia Maria Amorelli, Matteo Niutta, Stanislao Rizzo

**Affiliations:** * Ophthalmology Unit, Fondazione Policlinico Universitario A. Gemelli IRCCS, Rome, Italy;; † Università Cattolica del Sacro Cuore, Rome, Italy;; ‡ Ophthalmology Complex Operative Unit, Campus Bio Medico University, Rome, Italy; and; § Consiglio Nazionale delle Ricerche, Istituto di Neuroscienze, Pisa, Italy.

**Keywords:** macular surgery, myopic eyes, pars plana, trocar insertion, ultrasound biomicroscopy

## Abstract

**Purpose:**

To demonstrate through a diagnostic test used as a new preoperative assessment that trocar insertion for pars plana vitrectomy could be safely placed at a distance >4.0 mm in highly myopic eyes to facilitate the surgical maneuvers.

**Methods:**

Thirty eyes of 30 patients were tested with a biometer for the axial length measurement and with ultrasound biomicroscopy to measure the pars plana length. Pars plana lengths of highly myopic eyes were then compared with those of emmetropic eyes. The surgeon also measured the pars plana of highly myopic eyes intraoperatively and compared it with ultrasound measurements to assess ultrasound biomicroscopy reliability.

**Results:**

The mean axial length was 23.81 mm (SD ± 0.30) in the control group and 31.11 mm (SD ± 0.56) in the myopic group. The mean pars plana length was 4.96 mm (SD ± 0.19) in control eyes and 6.65 (SD ± 0.36) in myopic eyes. An extremely significant statistical difference (*P* < 0.001) was obtained by comparing the length of pars plana between control eyes and myopic eyes. The results of pars plana measurements were 6.65 mm (SD ± 0.36, ultrasound biomicroscopy) and 6.66 mm (SD ± 0.34, intraoperative measurements) in myopic eyes. The statistical comparison of the measurements in these two groups did not give a statistically significant result (*P* = 0.950).

**Conclusion:**

Ultrasound biomicroscopy is a reliable technique to calculate the length of pars plana in highly myopic eyes, where this parameter is significantly greater than that of emmetropic eyes. Trocars insertion for pars plana vitrectomy may be performed, in eyes with axial length >30 mm, in relative safety at a distance to limbus higher than 4 mm.

## Abstract 2

Supplemental Digital Content is Available in the Text.

Pars plana vitrectomy combined with internal limiting membrane peeling has been used since 2001 as a treatment for myopic macular disorders with or without retinal detachment.^1,2^ Pars plana vitrectomy is also considered the gold standard treatment for epiretinal membranes in myopic eyes, when symptomatic, with or without limiting membrane peeling peeling.^3^ Limiting membrane peeling dissection, however, in highly myopic eyes can be very challenging for several reasons. In particular, the axial length (AXL) >30 mm makes it difficult to reach the retinal surface with conventional 25/27-gauge (G) instruments, especially if a posterior staphyloma coexists.^4^ Being 25/27-G instruments <23 G, usually, they are not the first choice of use if the surgeon is dealing with high myopia eyes (>30 mm). For the same reason, it is complex to carry out a complete fluid-air exchange with 25/27-G instruments because of the impossibility of removing entirely the fluid from the posterior pole creating a “big issue” using heavy liquids.^5^

Furthermore, B-scan ultrasonography measurement before the vitrectomy was demonstrated to be useful to study the presence of the posterior myopic staphyloma and to measure the proper AXL trying to adopt the best surgical approach.^5^

In our experience, we have noticed that eyes affected by high myopia (AXL > 30 mm) usually have an anatomical alteration of the pars plana that appears to be longer than the average if compared with standard AXL (22–25 mm) eyes. From here, we postulated the concept of a “guided trocar insertion” of the dominant hand, more posterior to the standard 3.5 mm or 4.0 mm usually recommended to shorten the distance between the trocar (of the dominant hand) and the posterior pole and allowing the surgical tools to reach the posterior pole more effectively.

## Materials and Methods

**Fig. 1.**
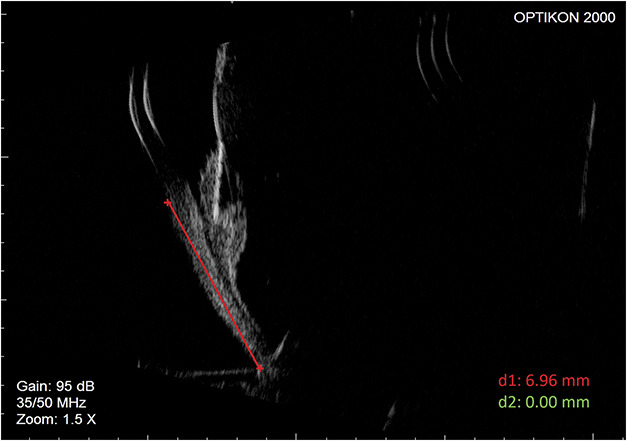
Example of ultrasound biomicroscopy (UBM) measurement of pars plana.

This study was conducted at the Catholic University of Sacred Heart of Rome in accordance with the Declaration of Helsinki and with the institutional Ethics Committee approval. All patients signed an informed consent. We evaluated 30 eyes, of which 15 control eyes had an AXL between 23 mm and 24 mm and 15 myopic eyes had an AXL >30 mm (Table 1). Myopic eyes were enrolled after indication for vitreoretinal surgery for a macular disorder (epiretinal membrane or macular hole or both). Eyes that underwent previous vitreoretinal surgery, intravitreal injections, or pathologic conditions able to affect the sclera (e.g., former scleral buckling for retinal detachment or eyes that had glaucoma filtering surgery) were excluded. Previous cataract surgery was not considered an exclusion criterion. Caucasian men and women between the ages of 55 years and 70 years were included in this study. In all eyes, the AXL measurement was performed using a biometer (IOL Master 500; Carl Zeiss Meditec, Jena, Germany), whereas pars plana in the superotemporal quadrant in right eyes and the superonasal quadrant in left eyes was measured through ultrasound biomicroscopy (AVISO-S; Quantel Medical, Lumibird Medical, Cedex, France) equipped with a 50-MHz probe (Figure 1). Measurements of the distance between the sclero-corneal limbus and the ora serrata (pars plana) of myopic eyes were then performed using the surgical caliper intraoperatively before proceeding with vitrectomy for macular surgery (Table 2). The measurements in these cases were calculated in the superotemporal quadrant in right eyes and the superonasal quadrant in left eyes and from the limbus, up to the ora serrata, which has been identified by the surgeon after scleral indentation using the O'Connor scleral depressor-marker at 10 o'clock and carried out with a caliper (see **Supplemental Digital Content 1**, http://links.lww.com/IAE/C148).

**Table 1.** Measurements of the AXL and the Nasal (Left Eyes) and Temporal (Right Eyes) Pars Plana in Control and Myopic Eyes

	R/L	Axial Length (mm)	Limbus/Ora Serrata Length—Nasal Side (mm)	Limbus/Ora Serrata Length—Temporal Side (mm)
Control eye 1	R	24.12		5.01
Control eye 2	R	23.87		5.25
Control eye 3	R	23.79		5.17
Control eye 4	R	24.08		5.12
Control eye 5	R	23.56		4.89
Control eye 6	R	24.15		5.21
Control eye 7	R	23.44		4.98
Control eye 8	R	23.92		5.08
Control eye 9	L	24.01	4.58	
Control eye 10	L	23.23	4.67	
Control eye 11	L	23.65	4.82	
Control eye 12	L	24.10	5.06	
Control eye 13	L	23.36	4.93	
Control eye 14	L	23.88	4.79	
Control eye 15	L	24.11	4.91	
Myopic eye 1	R	31.82		6.96
Myopic eye 2	R	31.23		6.32
Myopic eye 3	R	31.03		6.56
Myopic eye 4	R	30.58		6.06
Myopic eye 5	R	31.12		6.78
Myopic eye 6	R	30.23		6.21
Myopic eye 7	R	30.84		6.89
Myopic eye 8	R	31.20		6.61
Myopic eye 9	L	30.35	6.12	
Myopic eye 10	L	31.97	7.08	
Myopic eye 11	L	31.43	7.13	
Myopic eye 12	L	31.29	6.82	
Myopic eye 13	L	30.67	6.47	
Myopic eye 14	L	32.08	7.16	
Myopic eye 15	L	30.77	6.52	

L, left; R, right.

**Table 2.** Measurements of the AXL and Pars Plana Performed in Myopic Eyes With Ultrabiomicroscopy and Intraoperatively in Both Right and Left Eyes

	R/L Eye	Axial Length (mm)	Limbus/Ora Serrata Length—UBM (mm)	Limbus/Ora Serrata Length—Intraoperative Measurement (mm)
1	R	31.82	6.96	7
2	R	31.23	6.32	6.5
3	R	31.03	6.56	6.5
4	R	30.58	6.06	6
5	R	31.12	6.78	7
6	R	30.23	6.21	6.25
7	R	30.84	6.89	7
8	R	31.20	6.61	6.65
9	L	30.35	6.12	6.25
10	L	31.97	7.08	7
11	L	31.43	7.13	7
12	L	31.29	6.82	6.75
13	L	30.67	6.47	6.5
14	L	32.08	7.16	7
15	L	30.77	6.52	6.5

L, left; R, right; UBM, ultrabiomicroscopy.

An evaluation of the distribution shape of all measurements was performed through a frequency analysis. Measurements of pars plana in control eyes were then statistically analyzed and compared with those of myopic eyes. Regarding myopic eyes, we also compared the UBM measurements of pars plana with those obtained through the intraoperative measurement.

In both comparisons, we chose the Mann–Whitney test (nonparametric test equivalent to ANOVA), a statistical method appropriate when the independent variable is not normally distributed as for our data. A significance level (alpha) of 0.05 was chosen for all tests.

## Results

The mean AXL was 23.81 mm (SD ± 0.30) for nonmyopic eyes (control group) and 31.11 mm (SD ± 0.56) in myopic ones. The mean pars plana length was 4.96 mm (SD ± 0.19) in control eyes and 6.65 (SD ± 0.36) in myopic eyes. Based on the mean and SD of the measurements related to the pars plana in these two groups of eyes, a frequency analysis was processed. The analysis performed showed a more regular trend of data in control eyes compared with the group of myopic eyes, where the distribution did not result to be normal. The Mann–Whitney *U* test highlighted a statistically significance result between these two groups (*P* < 0.001).

The results of pars plana measurements were 6.65 mm (SD ± 0.36, UBM) and 6.66 mm (SD ± 0.34, intraoperative measurements using manual surgical caliper) in myopic eyes. For the intraoperative measurements too, a frequency analysis based on the mean and SD was calculated to evaluate their distribution. These data did not follow a normal distribution either.

This time, the Mann–Whitney *U* test showed that both the asymptotic significance and the exact significance (two-tailed) are >0.950 and thus above the significance level of 0.05. Therefore, no statistical difference between control and myopic groups can be determined with these data (Table 3).

**Table 3.** Difference in the Length of Pars Plana Measured With UBM (Blue) and Intraoperatively (Orange) in Myopic Eyes

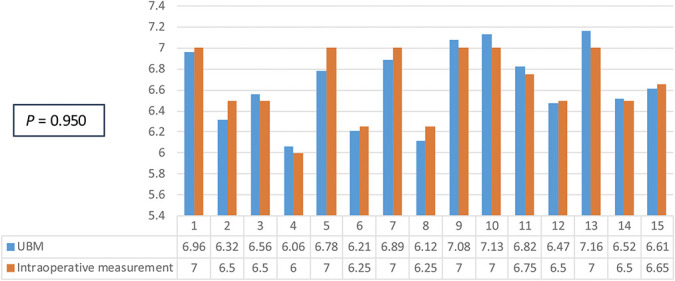

A not statistically significant result was found (*P* value = 0.950).

## Discussion

Vitrectomy in high myopic eyes is still a challenging surgery. Macular peeling, when indicated, in highly myopic eyes, is more difficult because of a different consistency of the MLI, which is thinner, more transparent, and easier to fragment into pieces during its removal.^6^ Additionally, the high AXL sometimes forces the surgeon to exert greater pressure on the scleral wall in an attempt to reach the macula and to make movements that facilitate the contact of the forceps and the hands with the lenses of the panoramic system, worsening the quality of intraoperative vision and increasing the surgical time.^7^

The greater distance between the macula and the trocars, in the case of high myopic eyes, forces the surgeon to widen the distance between the sclerotomies themselves to restore an appropriate working angle avoiding the excessive verticalization of the intravitreal instruments.^7,8^ Another possible approach is to use curved elongated instruments instead of straight ones^7^ or to remove trocars to directly insert the instruments through the sclera to gain a few millimeters.^9^ Recently, Al Sabti et al^4^ proposed the execution of a posterior scleral indentation through a Helvoston retractor to facilitate the peeling of MLI in eyes with AXL >34 mm and full-thickness macular hole. One limitation of this instrument is that it can be introduced only from the temporal side of the eye so that the surgeon is not able to peel with the dominant hand according to the laterality of the affected eye. Iwama et al showed that extending the limbus to cannula distance to 6.0 mm during pars plana vitrectomy to reach the posterior pole in eyes with AL >31 mm may be a safe procedure. The same authors also presented an interesting method to determine the distance between the cannula and the fovea and predict in which eyes the surgical instruments would not be able to reach the macula with a standard trocar's position of 3.5 mm from the limbus.^10^ In this regard, Hirono et al^11^ suggest the preoperative use of anterior segment optical coherence tomography to measure pars plana, comparing it with the intraoperative study of the pars plana to place safely the entry sites further than 4.0 mm to facilitate membrane peeling in highly myopic eyes. Similarly, our working group proposed a new method of preoperative evaluation of the pars plana using ultrasound biomicroscopy (UBM) in eyes with AXL >30 mm. The objective is to allow the insertion of the trocars safely at a distance from the limbus >4 mm to ensure the surgeon easier access to the posterior pole, overcoming the intraoperative technical difficulties of a highly myopic eye.

## Conclusion

The main contribution of this work is to show that the length of the pars plana in highly myopic eyes is significantly greater than that of emmetropic eyes and that this parameter can be reliably calculated by UBM. However, although this technique is not invasive for the patient, it is not available in all centers and it requires good experience for its use. With our results, nevertheless, we have shown that trocars insertion for pars plana vitrectomy may be performed, in eyes with AXL >30 mm, in relative safety and theoretically without carrying out any preoperative measurement at a distance to limbus higher than 4 mm. The main limitation of this study concerns the number of patients studied, and consequently, studies with a larger sample could give further validity and support to this technique to facilitate surgical maneuvers in highly myopic eyes.
